# Effect of age, sex and physiological stages on hematological indices of Banni buffalo (*Bubalus bubalis*)

**DOI:** 10.14202/vetworld.2016.38-42

**Published:** 2016-01-13

**Authors:** Mehul D. Patel, Abdul Lateef, Hemen Das, Ajay S. Patel, Ajay G. Patel, Axay B. Joshi

**Affiliations:** 1 Department of Physiology and Biochemistry, College of Veterinary Science & Animal Husbandry, Sardarkrushinagar Dantiwada Agricultural University, Sardarkrushinagar, Dantiwada-385506, Gujarat, India; 2 Department of Livestock Products Technology, College of Veterinary Science & Animal Husbandry, Anand Agricultural University, Anand- 388110, Gujarat, India

**Keywords:** age, banni, erythrocytic, lactation, leukocytic indices, pregnancy, sex

## Abstract

**Aim:**

To determine the physiological baseline values for hematological indices of Banni buffalo (*Bubalus bubalis*) as well as to assess their alteration due to age, sex and physiological stages.

**Materials and Methods:**

A total of 42 clinically healthy Banni buffaloes were categorized into seven groups (n=6): Group I (male calves ≤1 year), Group II (bulls >1 year), Group III (female calves ≤1 year), Group IV (pregnant lactating buffaloes), Group V (non-pregnant lactating buffaloes), Group VI (pregnant dry buffaloes), and Group VII (non-pregnant dry buffaloes). Blood samples collected aseptically from all the experimental groups were analyzed employing automated hematology analyzer. The data obtained were statistically analyzed; the mean and standard deviations were calculated and set as the reference values.

**Results:**

The erythrocytic indices *viz*. total erythrocytes count (TEC), hemoglobin, and packed cell volume (PCV) were significantly higher in bulls as compared to that of male calves unlike mean corpuscular volume, mean corpuscular hemoglobin (MCH), and MCH concentration. The female calves had higher TEC and PCV than the adult buffaloes irrespective of sex. The total leukocyte count (TLC) and neutrophil counts in male calves were significantly lower than the bulls unlike the eosinophil, while monocyte and basophil remained unchanged with age. The TLC, differential leukocyte count and platelet count varied non-significantly among the adult female groups at different physiological stages. However, neutrophils were found to be apparently higher in lactating buffaloes.

**Conclusion:**

The present study would be helpful for physiological characterization of this unique buffalo breed of Gujarat. Further, data generated may be a tool for monitoring the health and prognosis as well as diagnosis of diseases.

## Introduction

Blood indices are getting increasingly importance in veterinary medicine as indicators of the physiological, nutritional, metabolic, and clinical status of farm animals [1]. Hence, reference values for such indices become imperative for any disease diagnosis, prevention and controlling program as it forms the very basis for clinical interpretation of laboratory data [2]. It is well known that various factors *viz*. age, sex, breed and physiological stages affect the cellular hemodynamic [3,4]. However, pregnancy and lactation are the two most important stages in the life of dairy animals that affect metabolism resulting in alteration of the hematological parameters [5,6].

Although, various studies have been conducted on blood profile of buffaloes, the “Banni,” a unique indigenous breed of Gujarat, India remain untouched in this aspect. The breed is originated from the Banni area of Kutch district, and the total population in the state is about 5.25 lakhs, which is highest in Kutch followed by Sabarkantha, Surendranagar, Kheda and Banaskantha, respectively [7]. It is a black coat colored hardy breed with its characteristic inverted double coiled horns and known for its regular breeding cycle and high yielding capacity in harsh climatic conditions. The average milk yield is 10.05 kg/day when maintained under typical zero input and locally adapted production system in its breeding tract, which signifies productive potential of this unique germplasm [8]. The importance of “Banni” buffalo may also be gauged by the fact that it has been recognized as the 11^th^ buffalo breed of India by Indian Council of Agricultural Research, New Delhi. Further, with the ever-increasing interest in indigenous livestock breeds as a possible solution to increase efficiency of production in harsh conditions, there is an urgent need of basic physiological database on such breeds so as to plan and implement health and Disease Monitoring Program to ensure sustainable development of buffalo husbandry and economic viability of commercial buffalo farming in the country [9].

In view of above considerations, the present study was undertaken to determine reference values for complete blood count of clinically healthy Banni buffaloes as well as to study the influence of age, sex, pregnancy and lactation on different blood parameters of this breed.

## Materials and Methods

### Ethical approval

The present study was performed as a part of PG research work, which was approved by Director of Research, Sardarkrushinagar Dantiwada Agricultural University, Sardarkrushinagar, Gujarat.

### Place of study

The study was conducted between April 2014 and March 2015 at College of Veterinary Science and Animal House, Sardarkrushinagar in Banaskantha district of North Gujarat, India.

### Experimental animals

For the investigation, the experimental animals were selected from Cattle Breeding Farm, Bhuj. The region of the farm stretches between 22° to 24°N and 68° to 71°E and falls in the arid tract of Gujarat with the maximum annual average temperature of 39-45°C and 63% relative humidity.

About 42 clinically healthy animals of the farm were randomly categorized into six groups (n=6): Group I (male calves ≤1 year), Group II (bulls>1 year), Group III (female calves ≤1 year), Group IV (pregnant lactating buffaloes), Group V (non-pregnant lactating buffaloes), Group VI (pregnant dry buffaloes), and Group VII (non-pregnant dry buffaloes). All the buffaloes of the experimental groups were reared under standard feeding and husbandry conditions. The health of the selected animals was regularly examined based on behavior, rectal temperature, pulse rate, respiratory rate and fecal consistency. It is to be noted that since seven experimental groups were to be made at the same time as per age, sex, pregnancy and lactation, the sample size was to be restricted to 6 due to unavailability of more numbers of animals of each group in the farm during the experimental period.

### Collection of blood samples

About 10 ml of blood sample was collected aseptically from each animal of all the experimental groups by jugular vein puncture in vials containing tripotassium ethylene-diamine-tetra-acetic acid (P.H. Polyplast, Thane, India).

### Hematological analysis

Blood samples were analyzed for different hematological parameters *viz*. total erythrocyte count (TEC), hematocrit (HCT)/packed cell volume (PCV), hemoglobin (Hb), mean corpuscular volume (MCV), mean corpuscular hemoglobin (MCH), MCH concentration (MCHC), total leukocyte count (TLC), differential leukocyte count (DLC), and platelet/thrombocyte count (PLTs) using Automated Hematology Analyzer (Cell–Dyn 3700, Abbott Diagnostics, USA).

### Data analysis

The results were statistically analyzed using two-way ANOVA as per method of Snedecor and Cochran [10]. p<0.05 were considered to be statistically significant.

## Results

The mean±standard error values of erythrocytic indices, leukocytic indices and PLT counts of the experimental groups are presented in Tables-1-3, respectively. It was observed that the erythrocytic indices *viz*. TEC, Hb and PCV were significantly (p<0.05) higher in bulls as compared to that of male calves unlike MCV, MCH, and MCHC. The female calves had higher TEC (p<0.05) and PCV than the adult buffaloes irrespective of physiological stages. Conversely, adult females were found to have significantly (p<0.05) higher MCH than the calves. However, all the erythrocytic indices did not differ significantly between male calves and female calves, although TEC, Hb and PCV were apparently higher in female calves. Similarly, the TEC, Hb and PCV values of pregnant buffaloes were also non-significantly higher than those of non-pregnant buffaloes. Further, non-significant variation was also observed in the case of MCH, MCV and MCHC among adult female buffalo groups during different physiological stages.

**Table-1:** Erythrocytic indices of different experimental groups of Banni buffaloes.

Parameters	Male calves	Bulls	Female calves	Pregnant lactating buffaloes	Non-pregnant lactating buffaloes	Pregnant dry buffaloes	Non-pregnant dry buffaloes
TEC (M/µl)	9.47±0.64^b^	12.45±0.53^c^	10.18±0.56^b^	7.83±0.45^a^	6.98±0.32^a^	7.64±0.48^a^	6.76±0.37^a^
Hb (g/dl)	12.50±0.31^ab^	16.64±0.64^c^	13.36±0.76^b^	12.36±0.85^ab^	11.41±0.82^ab^	13.26±0.61^ab^	11.12±0.47^a^
PCV (%)	37.91±1.07^b^	44.68±1.24^c^	39.48±1.23^b^	34.05±1.39^a^	32.41±1.63^a^	35.83±1.26^ab^	33.60±1.70^a^
MCV (fl)	39.44±2.00	44.13±5.22	39.62±2.36	42.95±4.83	42.68±2.39	43.79±1.68	41.14±1.03
MCH (pg/cell)	14.84±0.68^abc^	14.19±0.63^ab^	13.28±0.79^a^	15.93±0.39^bc^	16.68±0.78^bc^	17.27±1.43^c^	17.13±0.76^c^
MCHC (g/dl)	35.67±2.09^a^	33.25±1.36^a^	35.99±1.31^a^	34.35±2.26^a^	33.60±1.41^a^	34.08±1.68^a^	36.82±1.89^a^

Means with same superscript within a row do not differ significantly from each other at 0.05% level of probability. TEC=Total erythrocytes count, Hb=Hemoglobin, PCV=Packed cell volume, MCV=Mean corpuscular volume, MCH=Mean corpuscular hemoglobin, MCHC=Mean corpuscular hemoglobin concentration

**Table-2:** Leukocytic indices of different experimental groups of Banni buffaloes.

Parameters	Male calves	Bulls	Female calves	Pregnant lactating buffaloes	Non-pregnant lactating buffaloes	Pregnant dry buffaloes	Non-pregnant dry buffaloes
TLC (K/μl)	8.16±0.64^a^	11.14±0.68^b^	8.95±0.75^a^	8.90±0.44^a^	8.16±0.38^a^	9.81±0.53^ab^	8.54±0.40^a^
Lymphocyte (%)	66.75±2.43	60.13±3.68	69.06±2.54	62.05±2.65	63.53±2.33	64.52±3.04	63.71±2.32
Neutrophil (%)	25.40±2.00^a^	34.01±2.64^b^	31.10±2.01^b^	34.35±1.92^b^	33.53±1.34^b^	30.93±1.50^b^	32.73±2.08^b^
Monocyte (%)	4.29±0.49^a^	4.55±0.63^a^	4.32±0.68^a^	4.87±0.73^a^	4.13±0.86^a^	4.58±0.59^a^	3.98±0.62^a^
Eosinophil (%)	3.74±0.71^a^	4.89±0.91^a^	3.33±0.87^a^	4.74±1.18^a^	4.13±0.38^a^	4.41±0.40^a^	4.22±0.88^a^
Basophil (%)	0.87±0.13^a^	0.85±0.34^a^	0.76±0.20^a^	0.70±0.06^a^	0.59±0.10^a^	0.67±0.09^a^	0.74±0.18^a^

Means with same superscript within a row do not differ significantly from each other at 0.05% level of probability. TLC=Total leukocyte count

**Table-3:** PLT count of different experimental groups of Banni buffaloes.

Parameter	Male calves	Bulls	Female calves	Pregnant lactating buffaloes	Nonpregnant lactating buffaloes	Pregnant dry buffaloes	Nonpregnant dry buffaloes
Platelet count (K/μl)	648.33±108.40^b^	522±92.59^ab^	587.83±91.84^ab^	485.67±75.78^ab^	432.17±52.21^ab^	390.33±28.73^a^	436.50±47.75^ab^

Means with same superscript within a row do not differ significantly from each other at 0.05% level of probability. PLT=Platelet

Table-2 indicates that TLC and neutrophil counts in male calves were significantly (p<0.05) lower than the bulls. The eosinophil counts of adult buffaloes were also numerically higher as compared with calves. On the contrary, lymphocyte and PLT counts were apparently higher in calves than adults, while monocyte and basophil remained unchanged with age. The TLC, DLC showed no significant variation among the adult female groups at different physiological stages. However, neutrophils were found to be higher in both the groups of lactating buffaloes than the non-lactating groups, while lymphocytes counts were relatively higher in young animals as compared to adults. Eosinophil count was apparently lower in female calves than male calves. Conversely, the pregnant buffaloes had numerically higher eosinophils compared to non-pregnant irrespective of lactation. Nonetheless, the values of eosinophil and monocyte count of the experimental groups were within the physiological range specified for buffalo.

Table-3 depicts that PLT count ranged from 390.33±28.73 (pregnant dry buffaloes) to 648.33±108.40 (male calves). However, the PLT count of male calves was non-significantly higher than that of bulls and female calves. The PLT count in female calves was numerically higher than the adult female buffaloes. Conversely, there was no significant difference between the groups of adult female buffaloes.

## Discussion

The importance of the indigenous gene pool of different livestock species has been acknowledged across the globe. In recent years, realizing the importance of indigenous buffalo germplasm for their high milk yield potential and ability to adapt in harsh agro-climatic condition of Gujarat, the breed improvement program on a massive scale is being implemented throughout the state. The enhancement of milk yield through improved management practices and effective disease prevention, control and treatment program have also been given priority. This denotes the significance of reference values on hematological indices as these are useful in determining the general health status of the animals [11] besides being an aid for differential diagnosis of clinical conditions as well as for monitoring response to therapy [12].

The present study revealed thatthe mean TEC, Hb and PCV of calves and adult buffaloes was in corroboration with Mohan *et al*. [13] in Murrah buffalo calves and Paul *et al*. [14] in adult buffaloes. However, relatively lower levels of these indices were recorded by Chandra *et al*.[15] in Murrah buffaloes at different ages. The mean values of MCV, MCH, and MCHC were found to be in accordance with to the reports of Haque *et al*. [16], Ellah *et al*. [17]. Nonetheless, significantly (p<0.05) higher TEC was observed in bulls as compared to male calves. This may be attributed to the role of androgens in enhancing the growth of erythroid progenitor cells in the presence of erythropoietin leading to higher rate of erythropoiesis, which in turn results in increased levels of Hb and PCV in bulls as observed in the current study [18]. Further, significantly (p<0.05) higher TEC of female calves than adult female buffaloes was in accordance to previous reports [19,20]. Similarly, significant alteration of TEC, Hb and PCV between male and female calves was also reported by Beechler *et al*. [11].

The pregnant buffaloes in both lactating and dry groups had apparently higher TEC, Hb and PCV as compared to the non-pregnant buffaloes of same groups. Higher levels of TEC in pregnant buffaloes were also reported by Patil *et al*. [21] unlike Kumar *et al*. [22], who recorded opposite trend. Similar to our findings, Mbassa and Poulsen [23] also recorded higher TEC in pregnant lactating animals than non-pregnant lactating and non-pregnant dry animals and concluded that this might be due to maternal adaptation to pregnancy in order to meet the requirements of growing fetus. Further, the fetal growth that occurs during pregnancy produces greater oxygen demands. This greater need for oxygen is compensated by the endocrine system that stimulates the release of erythropoietin by renal tissue [24]. The secretion of this circulating glycoprotein stimulates increased production of erythrocytes in the bone marrow resulting in hike of TEC during pregnancy [25]. Moreover, the apparently higher Hb and PCV value observed during pregnancy may be correlated with the higher TEC in this group of buffaloes. Kopp and Hetesa [26] and Chineke *et al*. [27] documented that high PCV reading may indicate an increase in the number of circulating RBCs. Nonetheless, the values of MCV, MCH, and MCHC did not vary significantly among experimental groups of adult female Banni buffaloes. Randhawa *et al*. [28] also reported that the values of MCV and MCH were not influenced by the lactation and dry stage in crossbred cows.

The circulating TLC generally represents the outcome of the dynamic production by bone marrow, the release of the cells to the peripheral circulation and their storage in different organs or pools. Sex differences in immune function are well-established in vertebrates [29]. As far as leukocytic indices of Banni buffaloes are concerned, it was observed that recorded data were comparable with those of earlier studies in different breeds of buffaloes [13,16,17,30,31]. The mean values of TLC and neutrophil counts were found to be significantly (p<0.05) higher in the bulls than the male calves, which were comparable with the study of Jacob [32], in which Gir bulls were found to have higher TLC than the male calves of different ages. However, higher lymphocyte count in calves than crossbred cows was reported by Shil *et al*. [33], which corroborates the finding of the current study. Whereas eosinophils were apparently higher in adult buffaloes than the calves, which were inconsistent with the study of Canfield *et al*. [34], who also found higher eosinophil counts in adult female buffaloes than female calves. The increase in a number of eosinophils recorded in the current study with the advancement of age might be an adaptive response of the body to various parasitic load and allergens to which it is exposed over a period of time. However, there was no obvious clinical sign of disease in experimental animals when they were sampled. Lactating buffaloes were found to have higher neutrophils than the dry buffaloes. The rise in neutrophil counts at lactation might be due to lactational stress leading to the release of endogenous corticosteroids [35]. The monocyte counts recorded in this study were in accordance with those of Ellah *et al*. [17] in heifers and Ali and Shukla [30] in normal cyclic post-partum buffaloes.

Current findings on PLT counts were in tune with those recorded by Das *et al*. [31] in lactating Mehsani buffaloes. Male calves hadnon-significantly higher PLT values than bulls, which was in line with the study of Mikniene *et al*. [36] in horses, where foals were found to have significantly higher PLT than the adults.

## Conclusion

It may be concluded that that age, sex and physiological stages alter hematological indices of Banni buffaloes. The data generated during the current investigation may be useful as reference values for the scientific community as this is the first study of its kind in this breed of buffalo. Further, it may assist the clinicians to assess the health status of buffaloes as well as in differential diagnosis of clinical conditions.

## Authors’ Contributions

AL and HD designed the experiment. MDP collected sample and carried out the experiment. HD and MP, ASP, AGP and ABJ prepared the manuscript. AL edited and revised the final draft of the manuscript. All authors read and approved the final manuscript.
